# Macrophage migration inhibitory factor as a therapeutic target in neuro-oncology: A review

**DOI:** 10.1093/noajnl/vdae142

**Published:** 2024-08-10

**Authors:** Jakub Jarmula, Juyeun Lee, Adam Lauko, Prajwal Rajappa, Matthew M Grabowski, Andrew Dhawan, Peiwen Chen, Richard Bucala, Michael A Vogelbaum, Justin D Lathia

**Affiliations:** Department of Molecular Medicine, Cleveland Clinic Lerner College of Medicine of Case Western Reserve University, Cleveland, Ohio, USA; Department of Cardiovascular and Metabolic Sciences, Lerner Research Institute, Cleveland Clinic, Cleveland, Ohio, USA; Department of Molecular Medicine, Cleveland Clinic Lerner College of Medicine of Case Western Reserve University, Cleveland, Ohio, USA; Institute for Genomic Medicine, Nationwide Children’s Hospital, Columbus, Ohio, USA; Department of Molecular Medicine, Cleveland Clinic Lerner College of Medicine of Case Western Reserve University, Cleveland, Ohio, USA; Department of Cardiovascular and Metabolic Sciences, Lerner Research Institute, Cleveland Clinic, Cleveland, Ohio, USA; Case Comprehensive Cancer Center, Cleveland, Ohio, USA; Rose Ella Burkhardt Brain Tumor and Neuro-Oncology Center, Neurological Institute, Cleveland Clinic, Cleveland, Ohio, USA; Department of Molecular Medicine, Cleveland Clinic Lerner College of Medicine of Case Western Reserve University, Cleveland, Ohio, USA; Department of Cardiovascular and Metabolic Sciences, Lerner Research Institute, Cleveland Clinic, Cleveland, Ohio, USA; Rose Ella Burkhardt Brain Tumor and Neuro-Oncology Center, Neurological Institute, Cleveland Clinic, Cleveland, Ohio, USA; Department of Molecular Medicine, Cleveland Clinic Lerner College of Medicine of Case Western Reserve University, Cleveland, Ohio, USA; Department of Cancer Biology, Lerner Research Institute, Cleveland Clinic, Cleveland, Ohio, USA; Section of Rheumatology, Allergy, and Immunology, Yale Cancer Center, Yale University School of Medicine, New Haven, Connecticut, USA; Department of Neuro-Oncology, H. Lee Moffitt Cancer Center and Research Institute, Tampa, Florida, USA; Department of Molecular Medicine, Cleveland Clinic Lerner College of Medicine of Case Western Reserve University, Cleveland, Ohio, USA; Department of Cardiovascular and Metabolic Sciences, Lerner Research Institute, Cleveland Clinic, Cleveland, Ohio, USA; Case Comprehensive Cancer Center, Cleveland, Ohio, USA; Rose Ella Burkhardt Brain Tumor and Neuro-Oncology Center, Neurological Institute, Cleveland Clinic, Cleveland, Ohio, USA

**Keywords:** glioblastoma, macrophage migration inhibitory factor, tumor microenvironment

## Abstract

Primary central nervous system (CNS) tumors affect tens of thousands of patients each year, and there is a significant need for new treatments. Macrophage migration inhibitory factor (MIF) is a cytokine implicated in multiple tumorigenic processes such as cell proliferation, vascularization, and immune evasion and is therefore a promising therapeutic target in primary CNS tumors. There are several MIF-directed treatments available, including small-molecule inhibitors, peptide drugs, and monoclonal antibodies. However, only a small number of these drugs have been tested in preclinical models of primary CNS tumors, and even fewer have been studied in patients. Moreover, the brain has unique therapeutic requirements that further make effective targeting challenging. In this review, we summarize the latest functions of MIF in primary CNS tumor initiation and progression. We also discuss advances in MIF therapeutic development and ongoing preclinical studies and clinical trials. Finally, we discuss potential future MIF therapies and the strategies required for successful clinical translation.

Each year, over 70 000 people in the United States are diagnosed with a primary central nervous system (CNS) tumor, and approximately 20 000 are diagnosed with a malignant tumor, with glioblastoma being the most common type.^1^ Despite persistent efforts by researchers and clinicians, patients continue to face a poor prognosis^2^; for this reason, there remains a significant need to better understand neuro-oncological diseases and develop effective therapies for patients. The initiation and progression of primary CNS tumors is a multifaceted process.^3^ Tumor cells possess distinct genetic and epigenetic alterations throughout tumorigenesis, while also exhibiting spatial heterogeneity. The tumor microenvironment (TME) is a dynamic ecosystem that shapes tumor behavior, with angiogenesis and neovascularization supplying nutrients to rapidly growing cells and immunosuppression reducing tumoricidal activity by leukocytes. Collectively, these processes promote tumor growth, and their mediators represent potential therapeutic targets.

Macrophage migration inhibitory factor (MIF) has been implicated in several of these oncological processes, including driving tumor cell proliferation, inhibiting apoptosis, increasing vascularization, promoting cell migration, and evading immune detection and destruction. Based on the importance of MIF in normal homeostasis and its alterations in pathological conditions such as cancer, there have been several classes of drugs developed against MIF. These therapeutic strategies have been tested in several preclinical models and early-phase clinical trials; however, these studies are limited in primary CNS tumor preclinical models and even more so in the clinical setting. The purpose of this review article is to report the latest information on MIF, its role in primary CNS tumors—with an emphasis on the most common malignant type, glioblastoma, but also including craniopharyngioma, medulloblastoma, meningioma, pituitary adenoma, and vestibular schwannoma—available MIF-directed treatments, and potential future therapies. MIF-directed treatments have the potential to complement existing therapies for primary CNS tumors, especially for malignant tumors such as glioblastoma and medulloblastoma. These new drugs may target existing MIF-mediated signaling pathways or act through novel mechanisms that remain to be identified in preclinical models. With its diverse and tissue-specific functions, MIF may drive additional aspects of tumorigenesis, such as enhancing tumor-promoting inflammation, activating invasion, and deregulating cellular metabolism. Potential new therapies may target one, or multiple, of these processes and expand the therapeutic arsenal against primary CNS tumors.

## Established Molecular Signaling Pathways

**Figure 1.**
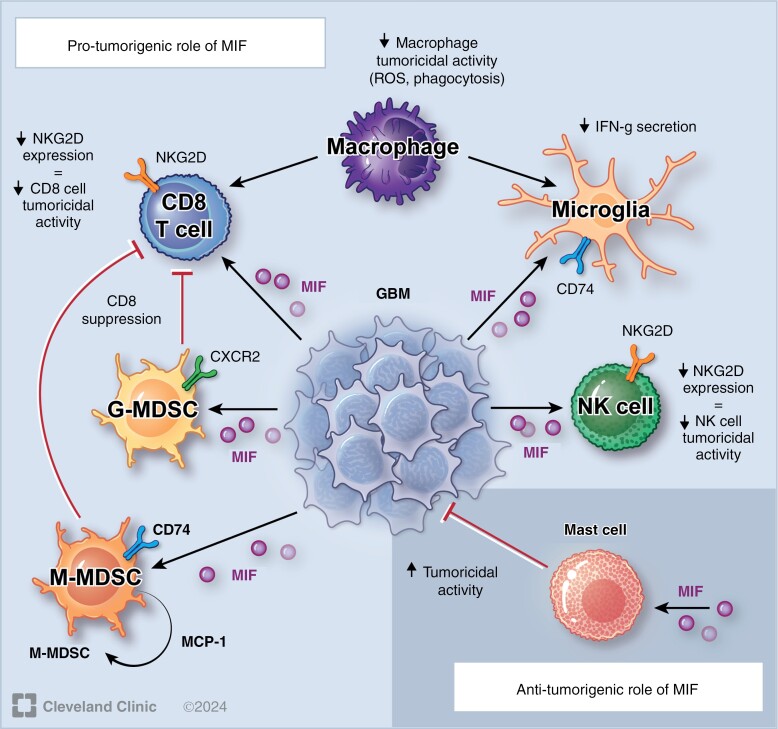
MIF-mediated signaling pathways in primary CNS tumors. CXCR2 = chemokine C-X-C motif receptor 2; G-MDSC = granulocytic myeloid-derived suppressor cell; GBM = glioblastoma; IFN-g = interferon gamma; M-MDSC = monocytic myeloid-derived suppressor cell; MIF = macrophage migration inhibitory factor; MCP-1 = monocyte chemoattractant protein-1; NK = natural killer cell; NKG2D = natural killer group 2 member D receptor.

Macrophage migration inhibitory factor is encoded in humans by the *MIF* gene located on chromosome 22q11.23.^4^ This highly conserved, homotrimeric protein is expressed by a wide range of cell types,^5^ including B and T lymphocytes,^6^ pituitary cells,^7^ vascular endothelial cells,^8^ adipocytes,^9^ and macrophages.^10^ Experimental models have uncovered several regulatory and effector pathways in MIF signaling.^11,12^ MIF is secreted by leukocytes in response to both inflammatory stimuli, such as lipopolysaccharide,^13^ and low concentrations of glucocorticoids.^14^ MIF functions as a proinflammatory cytokine in peripheral blood mononuclear cells, leading to increased levels of nuclear factor kappa B (NF-κB) to reduce glucocorticoid-induced immunosuppression.^15,16^ MIF results in decreased monocyte and macrophage expression of mitogen-activated protein kinase phosphatase-1 (MKP-1), increasing transcription of tumor necrosis factor alpha and interleukin 8 (I-8).^17^ These cytokines, as well as MIF, promote T lymphocyte proliferation and activation.^18–20^ MIF binds to a series of receptors, including CD74 and the chemokine C-X-C motif receptors (CXCR) CXCR2 and CXCR4 (Figure 1). The specific receptor functions and specificity are discussed in more detail below.

Macrophage migration inhibitory factor has been demonstrated to have enzymatic activity, functioning as a tautomerase to convert model substrates into their isomers.^21,22^ However, the physiological significance of these reactions remains undefined, and naturally occurring substrates have not yet been identified. Although the catalytic activity of the MIF tautomerase site is of uncertain significance, the catalytic region of the protein structure is important for cell signaling.^23^ The MIF tautomerase site binds to cluster of differentiation 74 (CD74), also known as major histocompatibility complex (MHC) class II invariant chain.^24^ CD74 is largely expressed in various immune cells, where it primarily serves intracellularly as a chaperone protein of MHC II for antigen presentation.^25^ MIF also affects intracellular signaling pathways that regulate B lymphocyte development and T lymphocyte thymic selection.^26,27^ In addition to leukocytes, CD74 expression has been identified in glioblastoma.^28^ Emerging studies have also described a role for MIF/CD74 signaling in malignant progression of low-grade glioma to high grade.^29^

In many cell types, the binding of MIF to CD74 recruits CD44 into a CD74 signaling complex, allowing Src tyrosine kinase to phosphorylate extracellular signal-regulated kinase 1 and 2 (ERK1/2).^24,30,31^ In neutrophils and macrophages, ERK1/2 phosphorylation decreases apoptosis, which increases leukocyte-mediated inflammation.^32^ In non-leukocytes, ERK1/2 phosphorylation increases podocyte and smooth muscle cell proliferation by modulating cell cycle-regulatory proteins, such as cyclin D1.^33,34^ Src also phosphorylates phosphoinositide 3-kinase (PI3K), potentiating Akt activity to reduce vascular endothelial cell apoptosis.^35^ In models of breast and endometrial cancer, MIF promotes angiogenesis through increased levels of vascular endothelial growth factor (VEGF) and IL-8.^36,37^ NF-κB, Src, and PI3K also upregulate adhesion molecules and mechanical proteins such as myosin to facilitate monocyte and dendritic cell migration.^35,38^ The CD74/CD44 complex also results in increased myeloid-derived suppressor cell (MDSC) secretion of monocyte chemoattractant protein-1 (MCP-1), which further recruits monocytes and MDSCs to the glioblastoma TME,^39^ and the phosphorylation of adenosine monophosphate-activated protein kinase (AMPK).^40^ AMPK is a critical regulator of cell metabolism, enhancing energy availability by acutely affecting catabolic and anabolic pathways and chronically augmenting mitochondrial biogenesis.^41,42^ These energetic changes may complement the inhibition of apoptosis to support tumor cell proliferation; however, this effect remains to be studied in primary CNS tumors.

Macrophage migration inhibitory factor also binds to chemokine receptors. In complex with CD74, CXCR2 is a G_αi_ protein-coupled receptor with a downstream signaling cascade that activates integrins, such as leukocyte function-associated antigen-1 (LFA-1), to anchor monocytes during their recruitment.^43^ The name for MIF, *macrophage migration inhibitory factor*, originates from its ability to affect chemokine receptor de-sensitization.^44,45^ MIF binding to CXCR4 alone has a similar effect on T lymphocyte recruitment.^43^ However, MIF does not inhibit leukocyte migration entirely. The leukocyte adhesion cascade requires initial, albeit brief, leukocyte arrest adjacent to the vascular endothelium prior to transmigration, after which leukocytes migrate toward their destination.^46^ Therefore, MIF was named based on the observation that it facilitates the first stage of the leukocyte adhesion cascade, that is, leukocyte arrest prior to transmigration. However, after transmigration, MIF acts through CXCR2 and CXCR4 to promote the movement of monocytes and T lymphocytes toward their target.^43^ These effects emphasize the diverse and context-dependent functions of MIF and its receptors.

Different cell populations express specific configurations of these receptors, enabling MIF to modulate a variety of pathways.^47^ CD74/CXCR4 regulates intracellular Akt signaling to promote leukocyte proliferation.^48^ MIF bound to atypical chemokine receptor 3 (ACKR3) alone increases platelet survival through this pathway,^49^ but in complex with CD74 and CXCR4, it activates ERK1/2 and zeta-chain-associated protein kinase-70 to drive migration of B lymphocytes.^50–52^ In MDSCs, monocytic MDSCs in the glioblastoma TME have higher levels of CD74 expression, while granulocytic MDSCs outside the CNS have higher expression of CXCR2.^39^

In neural stem progenitor cells, MIF binds to CD74, CD44, CXCR2, and CXCR4 in an unspecified configuration to increase cell proliferation and differentiation via the Wnt/β-catenin pathway.^53,54^ MIF also activates the signal transducer and activator of transcription 3 (STAT3)/SRY-box transcription factor 6 (SOX6)^55^ and paired box 6(PAX6)/chromatin helicase-DNA-binding protein 7 (CHD7)/translationally controlled tumor protein-1 (TPT1)^56,57^ pathways to promote neural stem progenitor cell proliferation. In a murine model of glioblastoma, TPT1 also promotes tumor cell proliferation.^57^ These signaling pathways are further augmented by the endocytosis of MIF and its direct binding with intracellular proteins, such as p53, in glioma-initiating cells.^58^ Other potential intracellular proteins may include c-Jun activation domain-binding protein 1 (Jab1),^59,60^ the NOD-, LRR-, and pyrin domain-containing protein 3 (NLRP3) inflammasome,^61^ and superoxide dismutase 1 (SOD1),^62^ which remain to be studied in primary CNS tumor models. In addition to influencing intracellular signaling, MIF also remodels the extracellular environment. MIF upregulates synovial fibroblast matrix metalloproteinase expression levels, which can promote tissue degradation and cell migration.^63^ In meningioma, co-expression of MIF with matrix metalloproteinase-9 was associated with increased tumor invasion and recurrence.^64^ In glioblastoma, MIF activates CXCR4 to promote a mesenchymal phenotype and contribute to tumor invasion.^65^

Macrophage migration inhibitory factor is a highly conserved protein expressed by numerous cell types, resulting in tissue-specific properties. It is primarily an inflammatory cytokine with variegated intra- and extracellular effects stemming from its diverse interactions. Its most notable functions are to reduce glucocorticoid-associated immunosuppression, decrease cell apoptosis, increase mitochondrial energy production, promote angiogenesis, and facilitate cell migration. These molecular pathways and their perturbations have important implications for primary CNS tumor development and progression.

## Impact on Primary CNS Tumor Development and Progression

Given the pleiotropic functions of MIF discussed above, there has been a concerted effort to characterize MIF expression in a variety of CNS tumors and link expression to tumor grade and outcome. Moreover, functional studies have also interrogated the multitude of cancer-related phenotypes associated with MIF, including cell survival/death, invasion, angiogenesis, and immune suppression.

**Figure 2.**
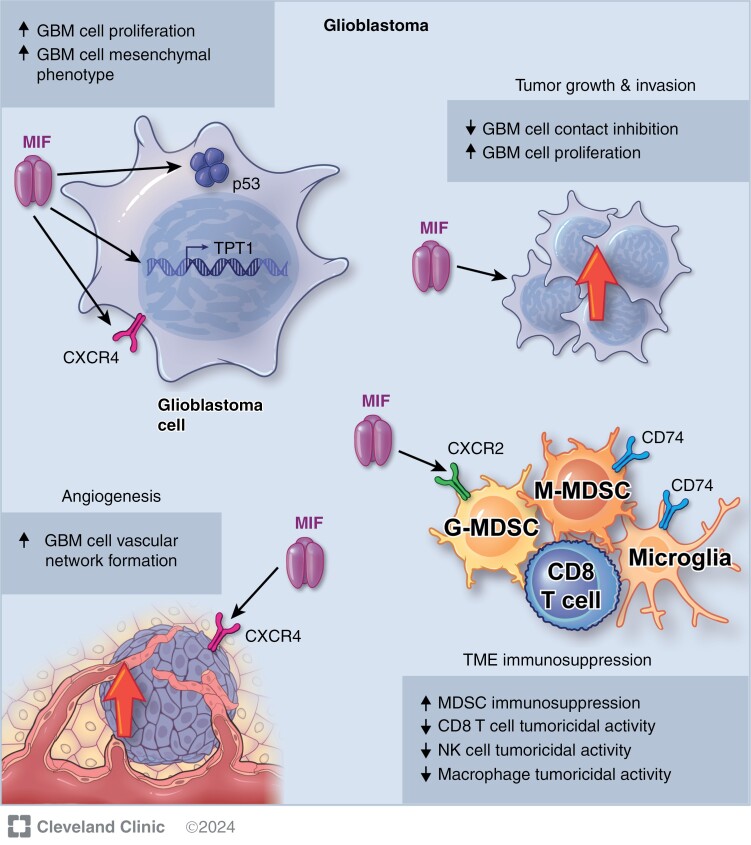
The effects of MIF on GBM behavior. CXCR2 = chemokine C-X-C motif receptor 2; CXCR4 = chemokine C-X-C motif receptor 4; G-MDSC = granulocytic myeloid-derived suppressor cell; GBM = glioblastoma; M-MDSC = monocytic myeloid-derived suppressor cell; MIF = macrophage migration inhibitory factor; MCP-1 = monocyte chemoattractant protein-1; NK = natural killer cell; TPT1 = translationally controlled tumor protein-1.

Macrophage migration inhibitory factor expression has been observed in several primary CNS tumor types. Pituitary adenoma expresses higher levels of MIF protein compared to nontumorous pituitary tissue, with consistently elevated levels across adenoma subtypes, such as nonfunctional or prolactin secreting.^66^ Single-cell ribonucleic acid (RNA) sequencing suggests that MIF secreted by pituitary adenoma cells and tumor-associated fibroblasts contributes to TME immunosuppression.^67^ Increased MIF protein expression has been also identified in meningioma^64,68^ and medulloblastoma.^69,70^ Craniopharyngioma demonstrates a wide range of MIF RNA levels that differ greatly among tumor cell subtypes based on single-cell sequencing, though there is limited information on the functional consequences of these expression patterns.^71^ Increased MIF protein levels have been implicated in several aspects of glioblastoma initiation and progression (Figure 2),^72–74^and many of these phototypes likely apply to other primary CNS tumors.

Macrophage migration inhibitory factor binds to p53 to both activate the cell cycle and inhibit apoptosis, which increases glioblastoma cell proliferation.^58^ These effects are consistent with its established function of decreasing leukocyte apoptosis. MIF also reduces contact inhibition to drive localized glioblastoma cell proliferation, though the exact mechanism remains unknown.^75^ By promoting a mesenchymal phenotype, MIF can increase glioblastoma cell growth more distally through tumor spread.^65^ However, in glioblastoma models, MIF was shown to be dispensable for tumor cell proliferation and survival, as shRNA-mediated reduction of MIF did not impact these phenotypes.^74^ This suggests that, in glioblastoma, the dominant function of MIF is likely not regulating cell proliferation or cell death.

Macrophage migration inhibitory factor also stimulates tumor vascularization. Hypoxia in necrotic regions of glioblastoma increases MIF secretion, which activates CXCR4 to induce peritumoral vascular network formation.^65,73^ This pathway represents a VEGF-independent mechanism of vascularization. However, studies suggest interdependent signaling between VEGF and MIF. The amount of MIF messenger RNA (mRNA) strongly correlates with VEGF mRNA levels in glioblastoma.^76^ This observation may stem from activation of a common upstream pathway; however, the addition of MIF protein induced VEGF secretion in an in vitro model of breast cancer.^36^ Conversely, an intracranial xenograft model of glioblastoma showed evidence of VEGF-dependent expression of MIF.^77^ This phenomenon remains understudied in primary CNS tumors, but existing evidence suggests that MIF and VEGF may reciprocally drive tumor angiogenesis.

Macrophage migration inhibitory factor has been observed to modulate AMPK activity and regulate energy production. This function may be implicated in primary CNS tumor cell proliferation. In nontumorous cells, MIF activates AMPK to increase energy production and reduce energy-intensive processes. However, in lung cancer cells, MIF overexpression inhibits AMPK activation to sustain tumor growth.^78^ This observation demonstrates the extensive effects of MIF, which are modulated by receptor heteromerization and their downstream signaling cascades. AMPK typically promotes mitochondrial degradation in response to energetic stress; however, glioblastoma cells alter mitochondrial availability and function to support cell proliferation.^79,80^ MIF may represent an additional pathway for increasing tumor cell energy production by inhibiting AMPK to decrease mitochondrial degradation.

In models of infectious diseases such as gram-negative endotoxemia or leishmaniasis, MIF functions as an inflammatory cytokine that promotes leukocyte activity.^13,81,82^ In medulloblastoma, MIF secreted by tumor cells did not directly promote T lymphocyte migration to the TME.^69^ However, MIF caused endothelial cells to secrete regulated upon activation, normal T-cell expressed, and secreted (RANTES) to attract T lymphocytes. Tumor-infiltrating T lymphocytes have been observed to have either pro- or anti-tumorigenic effects depending on their subtype, such as CD8^+^ or regulatory T lymphocytes. This MIF-mediated mechanism may represent one method for recruiting immunosuppressive leukocytes to the medulloblastoma TME. In the glioblastoma TME, MIF impairs leukocyte activity against tumor cells. Glioma cells secrete MIF, which binds to microglial CD74 to decrease interferon gamma release.^83^ This change inhibits anti-tumor macrophage polarization and contributes to an immunosuppressive TME. MIF additionally downregulates natural killer group 2 member D receptor (NKG2D) on both NK cells and CD8^+^ T lymphocytes to reduce their tumoricidal activity.^84^ Glioblastoma stem cells secrete MIF to activate CXCR2, prompting MDSCs to inhibit CD8^+^ T lymphocytes.^74^

CD8^+^ T lymphocyte inhibition may also occur through additional pathways, especially during the transformation from low- to high-grade glioma. In a murine model, the transition from low- to high-grade glioma was associated with decreasing CD8^+^ T lymphocyte infiltration, and both high- and low-grade glioma had increased protein expression of MIF and CD74 compared to nontumorous regions.^29^ Low-grade glioma macrophages had higher levels of CD74 protein expression, while high-grade glioma had increased cell surface expression of immunosuppressive galactin-3 and CD9. In human tissue, single-nucleotide polymorphisms in the *MIF* gene alter infiltration of CD8^+^ T lymphocyte, as well as that of other leukocytes, into the TME.^85^ In an in vitro murine model of neuroblastoma, a peripheral nervous system tumor, MIF overexpression resulted in overall (ie, CD3^+^) T lymphocyte overactivation and subsequent activation-induced apoptosis.^86^ This mechanism remains to be investigated in primary CNS tumors; however, it is possible that locally elevated levels of MIF in the TME may directly induce CD8^+^ T lymphocyte apoptosis. MIF also leads to impaired dendritic cell maturation and function, limiting immune surveillance in the glioblastoma TME.^87^ However, MIF retains some of its chemoattractive properties and promotes the recruitment of mast cells, which decrease glioblastoma cell proliferation by inhibiting STAT3.^88,89^ Greater mast cell levels have been associated with longer overall survival in patients with glioblastoma.^90^ It is likely that each immune cell type has a distinct role in the glioblastoma TME, which may be different in other primary CNS tumors. Further research on the diverse roles of tumor-infiltrating leukocytes can elucidate their contributions to the TME and impact on primary CNS tumor behavior.

The effects of MIF on glioblastoma development and progression extend to its prognostic utility. High MIF levels predict significantly reduced overall survival.^91^ In light of the diversity of MIF-mediated signaling pathways, this association is strengthened when coupled with increased CD74 expression by MDSCs,^39^ which have been identified as a significant contributor to the immunosuppressive TME in glioblastoma. MDSCs reduce T lymphocyte tumoricidal activity, increase immunosuppressive macrophage and regulatory T (Treg) lymphocyte counts, and decrease NK cell tumor cytotoxicity.^92^ The resulting immunosuppressive TME promotes glioblastoma progression and contributes to treatment resistance. Conversely, in craniopharyngioma, increased MIF protein levels have been associated with decreased tumor recurrence.^93^ However, there is limited information on the biological mechanisms underlying this observation. Importantly, craniopharyngioma represents a distinct disease from glioblastoma, which may explain these discordant findings. Craniopharyngioma is a benign tumor of embryonic origin located in the sellar and parasellar regions that affects both pediatric and adult patients.^94^ In contrast, glioblastoma is a malignant, cerebral tumor of neural and glial origin, with the highest incidence in older adults.^95^

It is critical to elucidate the mechanisms driving TME immunosuppression in primary CNS tumors, with the aim of targeting these processes and improving patient prognosis. Differential expression patterns of CD74 and CXCR2 by MDSCs within and outside the glioblastoma TME further emphasize that MIF signaling is location-specific. These differences can inform drug development to target TME-associated MDSCs and reduce their immunosuppressive signaling pathways. However, genetic differences introduce further variability in targeting MIF signaling. Certain genetic polymorphisms have been linked to greater CD8^+^ T lymphocyte infiltration in the glioblastoma TME, but significant associations with overall survival have not been demonstrated.^85^ In meningioma, tumor cell of chromosome monosomy 22 has been associated with decreased MIF gene expression and increased leukocytic tumor infiltration.^96^ Chromosome 22 is monosomic in meningioma and contains the neurofibromin 2 (*NF2*) gene, as well as the *MIF* gene. Epigenetic modifications may further regulate MIF gene expression and its function. Histone deacetylases and noncoding ribonucleic acids (ncRNAs) regulating the *MIF* gene have been implicated in gastrointestinal cancer initiation and progression.^97^ These features demonstrate the various genetic and epigenetic factors that can impact MIF gene expression and subsequent tumor behavior. Further research is needed to evaluate the potential role of genetic and epigenetic MIF regulation in primary CNS tumors.

In primary CNS tumors, MIF is implicated in several cancer hallmarks, including resisting cell death, sustaining proliferative signaling, accessing vasculature, and avoiding immune destruction.^98,99^ A potential role in further hallmarks and their relationships to MIF remains to be studied, including deregulated cellular metabolism. Given the importance of MIF in driving many tumor-accelerating phenotypes, MIF represents a promising therapeutic target for primary CNS tumors. Specifically, the identification of strategies that impact multiple pro-cancer phenotypes regulated by MIF could result in a more effective therapeutic response. Given the current data in glioblastoma suggesting a tumor cell-extrinsic function for MIF, it is likely that targeting MIF may be more successful by impacting the TME (immune suppression, angiogenesis) rather than the tumor cells themselves, and this should be explored alone and in combination with known immune modulators and antiangiogenic agents.

## MIF As a Therapeutic Target in Primary CNS Tumors

Targeting MIF has been an efficacious strategy in a variety of preclinical models of cancerous and noncancerous diseases.^100,101^ These compounds act through various mechanisms, including decreasing MIF assembly from its protein subunits, blocking MIF from binding to its target transmembrane receptors, and reducing MIF intracellular activity. These drugs are currently being, or have the potential to be, translated to treat primary CNS tumors. There is activity both in preclinical models and early-phase clinical trials.

*N*-acetyl-p-benzoquinone (NAPQI) is a metabolite of acetaminophen that covalently binds to the MIF tautomerase site (ie, orthosteric) (Table 1).^102^ This modification impairs MIF binding to extracellular receptors and its subsequent signaling activity. These mechanistic effects were observed in vitro with clinical doses of acetaminophen. This approach demonstrated the efficacy of targeting MIF through its tautomerase site, which has limited physiological catalytic activity but important effects on cell signaling. This mechanism has been also identified for plant-derived metabolites oxidized (-)-epicatechin-quinone (oxEC)^103^ and sulforaphane.^104^ Sulforaphane administration induced apoptosis in immunosuppressive MDSCs and several glioblastoma cell lines in vitro,^105–107^ and a similar effect has been observed in medulloblastoma^108^ and vestibular schwannoma.^109^ In vivo studies found that sulforaphane decreased tumor volume growth in murine models of vestibular schwannoma and glioblastoma.^109,110^ Perhaps more importantly, these findings informed the search for synthetic compounds. (*S,R*)-3-(4-hydroxyphenyl)-4,5-dihydro-5-isoxazole acetic acid methyl ester (ISO-1) was first synthesized as a reversible, competitive inhibitor of the MIF tautomerase site.^111^ ISO-1 demonstrated dose-dependent effects on arachidonic acid secretion, dexamethasone regulation, and glioblastoma cell proliferation.^112,113^ Specifically, ISO-1 reduced in vitro glioblastoma cell growth and increased CD74 protein expression. It also decreased tumor growth in vivo while reducing the number of immunosuppressive macrophages in the TME.^114^ Analogues of ISO-1 have been developed in an effort to further refine the efficacy of this drug class. 3-(2ʹ-methylphenyl)-isocoumarin (SCD-19), CPSI-1306, CPSI-2705, and ISO-66 have been tested in preclinical models of bladder, colorectal, lung, and skin cancer,^115–117^ whereas 3-(3-hydroxybenzyl)-5-methylbenzooxazol-2-one (Debio1036) and OXIM-11 have been investigated in non-oncological diseases such as arthritis and sepsis.^118–121^

**Table 1.** A Summary of MIF-Directed Treatments and Their Preclinical Efficacy Against Primary CNS Tumors

Mechanism of Action	Name	Reported Efficacy Against Primary CNS Tumors	Development Status Against Primary CNS Tumors
Small-molecule inhibitors: orthosteric inhibitors (ie, binding to the MIF tautomerase site)	2-OPB	Not reported	Not tested
	4-IPP	Glioblastoma In vitro (murine)^126^	Preclinical
	CPSI-1306	Not reported	Not tested
	CPSI-2705	Not reported	Not tested
	Debio1036/MIF098	Not reported	Not tested
	ISO-1	Glioblastoma In vivo (murine)^114^	Preclinical
	ISO-66	Not reported	Not tested
	NAPQI	Glioblastoma In vitro^102^	Preclinical
	oxEC	Not reported	Not tested
	OXIM-11	Not reported	Not tested
	SCD-19	Not reported	Not tested
	Sulforaphane	Glioblastoma In vivo (murine)^109,110^ Medulloblastoma In vitro^108^ Vestibular schwannoma In vivo (murine)^109^	Preclinical
Small molecule inhibitors: allosteric inhibitors	Iguratimod	Glioblastoma In vivo (murine)^159^	Preclinical
	p425	Not reported	Not tested
	L2-4048 (chimeric molecule of ibudilast and p425)	Not reported	Not tested
Small-molecule inhibitor: MIF destabilization (ie, trimer formation disruption, chaperone loss-mediated degradation)	Ebselen	Glioblastoma In vitro^162,163^ Vestibular schwannoma In vitro^164^	Preclinical
	Tanespimycin	Glioblastoma In vitro^167,168^	Preclinical
Peptide drugs: ligand–receptor binding with CD74	C36L1	Not reported	Not tested
	DRα1-MOG-35–55	Not reported	Not tested
	RTL1000	Not reported	Not tested
Peptide drugs: ligand–receptor binding with CXCR2	MIF-(40-49)	Not reported	Not tested
	MIF-(47-56)	Not reported	Not tested
Monoclonal antibody: anti-MIF monoclonal antibodies	BaxB01	Not reported	Not tested
	BaxG03	Not reported	Not tested
	BaxM159	Not reported	Not tested
	Imalumab	Not reported	Not tested
	ON104	Not reported	Not tested
	ON203	Not reported	Not tested
Monoclonal antibody: anti-CD74 monoclonal antibody	Milatuzumab	Not reported	Not tested

2-oxo-4-phenyl-3-butanoate (2-OPB) is a synthetic compound that covalently binds to the MIF tautomerase site, resulting in irreversible MIF inhibition.^122^ Although this compound has not been tested in any disease models, this mechanism has been identified for other compounds, namely 4-iodo-6-phenylpyrimidine (4-IPP).^123^ 4-IPP has a similar efficacy to ISO-1, but the potency of 4-IPP is approximately 10 times greater based on kinetic studies, enabling a comparable effect at a much lower dose. 4-IPP impairs MIF both intra- and extracellularly.^124,125^ Intracellularly, 4-IPP prevents MIF from binding to chaperone protein p115, which decreases MIF secretion. Extracellularly, 4-IPP blocks MIF from binding to CD74, which reduces the downstream signaling activity of MIF through this transmembrane receptor. In a murine flank model of glioblastoma, 4-IPP increased cancer stem cell apoptosis and reduced post-radiation therapy mesenchymal transdifferentiation.^126^ However, these effects are difficult to ascribe directly to MIF, as 4-IPP also inhibits the MIF homologue D-dopachrome tautomerase.^127^ Nevertheless, the demonstrated efficacy of 4-IPP warrants further investigation. More broadly, many orthosteric MIF inhibitors have not been tested in primary CNS tumor models. These compounds represent potential new treatments for primary CNS tumors, as well as scaffolds for the development of refined analogues with improved efficacy and potency and decreased toxicity.

Ibudilast is a small-molecule inhibitor of MIF and several phosphodiesterases, primarily types 1–4.^128–130^ Specifically, ibudilast binds adjacent to the MIF tautomerase site, resulting in allosteric inhibition. First approved in Japan to treat asthma, its indications have been expanded to post-stroke dizziness and allergic conjunctivitis.^131–133^ Ibudilast crosses the blood-brain barrier (BBB), making it a promising treatment for neurological diseases.^134^ In multiple sclerosis, a randomized Phase 2 trial found no significant benefit for reducing active lesion formation.^135^ However, a more recent Phase 2 trial found ibudilast to be associated with a significantly slower rate of brain atrophy.^136^ In amyotrophic lateral sclerosis, 2 early-phase trials evaluated its safety and tolerability in this patient population.^137,138^ These findings have informed the design of an ongoing Phase 2/3 trial studying the effects of ibudilast on patient functional status and survival.^139^ Ibudilast has been also studied in glioblastoma, where it increased CD8+ T lymphocyte frequency in the TME, but not in nontumorous brain tissue or peripheral blood, in a murine model.^39^ In a separate preclinical study, ibudilast combined with temozolomide increased tumor cell death in vitro and prolonged overall survival in vivo.^140^ Based on these results, ibudilast is currently being investigated in combination with temozolomide in a Phase 1/2 trial for newly diagnosed and recurrent glioblastoma (Table 2).^141^

**Table 2.** A Summary of MIF-Directed Treatments and Their Clinical Trial Status

Mechanism of Action	Name	Reported Efficacy Against Primary CNS Tumors	Development Status Against Primary CNS Tumors
Small-molecule inhibitors: allosteric inhibitors	Ibudilast	Glioblastoma In vivo (murine)^140^	Phase 1/2 clinical trial (ongoing)

Chicago Sky Blue 6B (p425) is a dye identified in a screen for allosteric MIF inhibitors.^142^ p425 binds 2 separate MIF proteins, occupying their tautomerase sites to inhibit their interactions with CD74. p425 is also thought to reduce the proinflammatory effects of MIF by impacting additional functional regions beyond its tautomerase site, such as its thiol-protein oxidoreductase site. Although not studied in primary CNS tumors, p425 decreased colorectal carcinoma cell migration in vitro, and MIF administration increased cancer metastasis in vivo.^143^ L2-4048 is a chimeric molecule composed of ibudilast and the tautomerase site-binding segment of p425.^144^ Initial mechanistic evaluation supports improved activity against MIF due to both its inhibition of the MIF tautomerase site and its promotion of MIF-MIF binding, which sequesters MIF from binding to its targets. L2-4048 also addresses the large size of p425 while maintaining its functional portion. Chimeric compounds may represent the next generation of MIF targeted therapies; however, these compounds remain to be studied in preclinical models of primary CNS tumors.

Iguratimod is an additional allosteric small-molecule inhibitor of MIF.^145^ Initially developed as an anti-inflammatory agent, it was found to have a distinct mechanism of action compared to classical non-steroidal anti-inflammatory drugs (NSAIDs).^146–149^ In addition to reducing cyclo-oxygenase-2 (COX-2) activity,^150^ iguratimod reduces NF-κB activation,^151,152^ IL-17-mediated proinflammatory pathway activity,^153^ B-lymphocyte cytokine synthesis,^154^ and MIF signaling activity.^155,156^ Iguratimod has been approved as a disease-modifying anti-rheumatic drug (DMARD) for rheumatoid arthritis in China^157^ and Japan.^158^ However, iguratimod has been studied in other diseases, such as glioblastoma. Iguratimod-loaded nanoparticles significantly decreased tumor cell proliferation in vitro and tumor volume in vivo in a flank murine model of glioblastoma.^159^ Although these results are promising as a single-agent treatment, the efficacy of iguratimod may be improved by combining it with other drug classes, such as immune checkpoint inhibitors based on the principle that iguratimod can reduce local immune suppression which could then enhance the efficacy of immune activating approaches. In a murine model of lung cancer, combined treatment with iguratimod-loaded nanoparticles and programmed cell death protein-1 (PD-1)-expressing nanovesicles decreased lung cancer cell proliferation and migration in vitro and tumor volume in vivo.^160^ Synergistic combinations of iguratimod require investigation against primary CNS tumors to determine their possible translation to clinical trials. Additionally, the design of clinical trials investigating combination therapies can greatly benefit from a rational drug selection strategy. Selecting medications that act through distinct mechanisms can collectively target multiple oncogenic pathways in parallel, promoting drug synergy. Synergistic drug combinations can reduce the required amount of each medication, which can possibly address issues of single-agent drug toxicity. Drug repurposing is also a promising strategy. The evaluation of medications currently used in clinical practice, either in the United States or abroad, can provide a large amount of pharmacokinetic data that can inform clinical trial design. For iguratimod, its international use for over a decade offers significant information on its safety and efficacy, and the addition of the anti-PD1 monoclonal antibody pembrolizumab, a Food and Drug Administration (FDA)-approved therapy for select solid tumors, may further accelerate clinical trial initiation for patients with primary CNS tumors.

Macrophage migration inhibitory factor is a homotrimer, making its oligomerization an additional target. Ebselen is a synthetic, selenium-based antioxidant that is also a trimer-disrupting inhibitor of MIF.^161^ Ebselen has been found to induce the dissociation of MIF into its monomers, which disrupts the tautomerase site found within the MIF trimer. Ebselen has been observed to promote in vitro glioblastoma cell apoptosis and decrease migration, as well as to decrease inflammatory cytokine secretion.^162,163^ Conversely, a cell line of vestibular schwannoma was modified to increase intracellular reactive oxygen species, resulting in increased apoptosis, and ebselen treatment rescued tumor cells from apoptosis.^164^ These contrasting results—that is, ebselen administration increasing glioblastoma cell apoptosis but decreasing vestibular schwannoma apoptosis—likely stem from the multimechanistic effects of ebselen as an antioxidant and MIF trimer disruptor. In addition to targeting MIF oligomerization, there have been medications tested that inhibit chaperone proteins from protecting MIF against degradation. Tanespimycin (17AAG) is an antibiotic-derived compound that inhibits heat shock protein 90 (HSP90) from functioning as a MIF chaperone protein, resulting in increased MIF degradation.^165,166^ Tanespimycin administration has been found to increase glioblastoma cell apoptosis in vitro.^167,168^ Modifications such as micelle nanocarrier delivery demonstrated even higher efficacy and potency.^169^ However, acquired resistance has been observed, which may require next-generation analogues or synergistic combination treatments to overcome this challenging adaptation by glioblastoma cells.^170–174^ Additionally, targeting HSP90 may invoke many off-site effects due to its widespread effects on the function of numerous kinases, steroid receptors, and transcription factors.^175^ Overall, MIF trimer destabilization is an additional avenue for reducing MIF activity by targeting MIF availability upstream of ligand–receptor interactions. This class of MIF inhibitors requires further preclinical investigation, both as a single agent and in combination with existing treatments to evaluate drug synergy.

In addition to small-molecule inhibitors, protein-based treatments have been developed against MIF, including protein subunits (ie, peptides) and monoclonal antibodies. DRα1-MOG-35–55 is a human leukocyte antigen-DR (HLA-DR)-derived peptide that binds CD74 and prevents its interaction with MIF.^176^ This function was able to reduce MIF signaling in a murine model of multiple sclerosis. Similarly, recombinant T-cell receptor ligand-1000 (RTL1000) is an HLA-DR-derived peptide that binds CD74.^177^ RTL1000 has been studied in a Phase 1 clinical trial for multiple sclerosis, demonstrating favorable safety and tolerability.^178^ These results may inform preclinical and clinical studies in primary CNS tumors. C36 V_L_ immunoglobulin complementary determining region-1 (C36L1) is a peptide developed to investigate adjuvant immune system stimulation against various cancer types.^179^ C36L1 demonstrated promising efficacy against human and murine melanoma in vitro. Specifically, C36L1 binds to CD74 to inhibit MIF signaling, which promotes the anti-tumorigenic activity of dendritic cells, macrophages, and T lymphocytes while reducing the immunosuppressive activity of regulatory T cells and MDSCs in the TME.^180^ These findings have the potential to be translated to primary CNS tumors. Beyond CD74, peptide inhibitors have been developed to target additional extracellular protein domains that normally interact with MIF. Both MIF-(40-49) and MIF-(47-56) are peptides that block the interaction between the MIF N-like loop and the CXCR2 receptor N domain, exoloop EL1, and exoloop EL2 in an in vitro model of atherosclerosis.^181^ Increased CXCR2 activation has been associated with the progression of several cancer types, including breast, lung, and pancreatic cancer.^182–184^ This pathway, and its inhibition with peptide therapies, requires further investigation in primary CNS tumors.

Several monoclonal antibodies have been developed against MIF. BaxB01, BaxG03, and BaxM159 have demonstrated increased prostate cancer cell apoptosis in vitro by inhibiting ERK1/2 signaling, resulting in decreased tumor growth in vivo in a xenograft murine model.^185,186^ Imalumab (Bax69) has been studied in a Phase 1 clinical trial that found promising efficacy against solid tumors such as colorectal carcinoma, non–small-cell lung cancer, and ovarian cancer, with one-third of patients overall exhibiting stable disease.^187^ Of note, patients with primary or metastatic brain tumors were excluded from this trial, which prevented the investigation of the efficacy of imalumab within the CNS. Imalumab has also informed the development of second-generation monoclonal antibodies. ON203 demonstrated a prolonged half-life and improved pharmacokinetic profile compared to imalumab in a xenograft murine model of prostate cancer.^188^ ON104 has been tested in a murine model of rheumatoid arthritis, but its efficacy against tumors in the CNS remains unknown.^189^ Milatuzumab is a monoclonal antibody that binds CD74 to inhibit its interaction with MIF.^190^ Preclinical studies demonstrated antitumoral efficacy in animal models of hematological cancers.^190,191^ These studies have been followed by several early-phase clinical trials, leading to its orphan approval for multiple myeloma and B cell malignancies.^192–195^ Overall, with their documented safety and tolerability, monoclonal antibodies represent additional anti-MIF therapies that may be repurposed against primary CNS tumors such as glioblastoma. Alternatively, their sequences may be modified to generate future-generation antibodies, or their segments may be explored to develop nanobody therapies.^196^ Future research must also consider BBB penetrance, which remains a significant challenge for protein-based treatments.

As discussed above, there have been many MIF-targeting strategies developed that are at various stages in preclinical testing and clinical evaluation, some of which are in primary CNS tumors. While many of the treatments described above (including small molecule inhibitors, chimeric compounds, peptide inhibitors, monoclonal antibodies, and nanobodies) have been developed for both cancerous and noncancerous diseases, the treatment of primary CNS tumors presents several major challenges to drug development. Foremost is the ability for a given therapy to reach brain tissue in sufficient concentrations to induce a meaningful effect, if the intended mechanism of action is modulating MIF locally in the brain. Crossing the BBB and blood-tumor barrier remains a challenge for many therapeutic strategies, including small molecules and antibodies. That is, while the BBB and blood-tumor barrier can be regionally leaky, the ability to achieve high concentrations in the brain is challenging. Therefore, prioritizing brain penetrant inhibitors (such as ibudilast and those that have been developed for and tested in neurological disorders like multiple sclerosis) for preclinical investigation will likely maximize the chances of success in early-phase clinical trials. The second challenge for the use of MIF-targeting agents in primary CNS tumors is mitigating neurological off-target effects to reduce toxicity. Neurological side effects may be idiosyncratic and challenging to predict, representing a major challenge in CNS drug development. While these effects can be somewhat mitigated through an in-depth understanding of MIF signaling in multiple neural cell types as controls during early-phase therapeutic development efforts, global neurological effects, and particularly the effects when MIF-targeting is combined with chemotherapy and/or radiotherapy, may only be uncovered through human or primate studies. Therefore, MIF targeting and dosing strategies that have accounted for neural function should also be prioritized.

## Conclusions and Future Perspectives

Foundational studies have demonstrated that high levels of MIF have been associated with a poor prognosis in several primary CNS tumors. MIF has been shown to contribute to tumor initiation and progression by decreasing cell apoptosis, increasing angiogenesis, driving cell migration, and promoting immunosuppression. As MIF is an inflammatory cytokine that has diverse, tissue-specific functions, its function across primary CNS tumors is likely to also show some diversity, necessitating a careful assessment of both MIF expression and function in each tumor type. When possible, careful assessment of MIF and its known receptors in human samples should be integrated with cell responses and preclinical models to lay the groundwork for translational studies that could use both patient samples and assessment of MIF-targeting strategies in preclinical models. Importantly, there are numerous treatments available against MIF, including small-molecule inhibitors, peptide inhibitors, and monoclonal antibodies. While many of these treatments have been developed in tumor models, they remain to be studied in many primary CNS tumor types. Continued evaluation of these therapies in preclinical and clinical studies must consider several key tumor-specific considerations, including BBB penetrance and the impact on both the TME (including immune cells) and normal neural cells. Once benchmarking studies have been performed in preclinical models as single agents, follow-up assessments of combination approaches including standard of care (radiation, chemotherapy), as well as immune therapies and antiangiogenic approaches, are likely to help provide guidance for the next set of early-phase clinical trials for CNS tumors. In addition, integration of genetic and epigenetic data may identify patients with polymorphisms most likely to respond to anti-MIF treatments, highlighting the possible impact of pharmacogenomics on treatment selection. Ultimately, MIF represents a promising therapeutic target in neuro-oncology and has the potential to inform the development of new therapies for patients with both primary and metastatic CNS tumors.
